# Innovative nucleic acid detection of *Clostridioides difficile* utilizing the PAM-unconventional, one-step LAMP/CRISPR-Cas12b detection platforms

**DOI:** 10.3389/fcimb.2025.1594271

**Published:** 2025-05-29

**Authors:** Yizhuo Zhang, Luqin Lv, Su Xu, Yijian Chen

**Affiliations:** Institute of Antibiotics, Huashan Hospital, Fudan University & Key Laboratory of Clinical Pharmacology of Antibiotics, National Health Commission, Shanghai, China

**Keywords:** *Clostridioides difficile*, LAMP, CRISPR-Cas12b, nucleic acid detection, point-of-care testing

## Abstract

**Introduction:**

*Clostridioides difficile* (*C. difficile*), a human pathogen that causes diarrhea and colon lesions, has garnered widespread attention. Rapid and accurate detection of bacterial virulence factors is essential for the diagnosis of *C. difficile* infection (CDI). To date, numerous laboratory tests have been developed; however, none fully meet the combined requirements of speed, cost-effectiveness, portability, sensitivity, and specificity. Molecular diagnostic technologies based on CRISPR-Cas systems have provided a promising solution to this challenge. Nonetheless, the limited compatibility between pre-amplification and CRISPR cleavage, coupled with the inherent selectivity of CRISPR systems for protospacer adjacent motif (PAM) sequences near the target site, poses additional constraints on the broader adoption of this approach.

**Methods:**

Here, we developed PAM-unconventional, One-step LAMP/CRISPR-Cas12b (POLC) detection platforms for the toxin-encoding genes *tcdA* and *tcdB* of *C. difficile*.

**Results:**

The POLC platforms operated at 60 °C, enabling result interpretation either through fluorescence intensity measurements or direct visualization under UV light. The limits of detection (LoDs) ranged from 3 to 14 copies/μL using a fluorescence reader and from 6 to 18 copies/μL via direct observation. Compared to qPCR, which typically requires over an hour, the POLC platforms reduced the detection time to approximately 40 minutes. Each reaction cost approximately USD 6.5, offering a substantial cost saving compared to qPCR-based commercial kits (over USD 10 per test). In clinical validation with 55 fecal samples, the *tcdA* POLC assay achieved 86.4% sensitivity and 84.8% specificity, while the *tcdB* POLC assay demonstrated 96.6% sensitivity and 100% specificity, using qPCR as the reference standard.

**Discussion:**

Our research presents innovative CRISPR-based one-step nucleic acid detection platforms that eliminate canonical PAM sequence requirements. These platforms exhibit high sensitivity and specificity while achieving rapid detection under simple conditions, making them promising candidates for clinical diagnostics and point-of-care testing (POCT).

## 1 Introduction

*Clostridioides difficile* (*C. difficile*) is a Gram-positive, anaerobic bacterium responsible for both nosocomial and community-associated gastrointestinal infections (Smits et al., 2016; Guh and Kutty, 2018). *C. difficile* infection (CDI) typically presents with diarrhea and may progress to severe complications, such as ileus, pseudomembranous colitis, and colon necrosis (Smits et al., 2016; McDonald et al., 2018). In 2019, the Centers for Disease Control and Prevention classified CDI as one of the most serious “urgent threats” to public health (Centers for Disease Control and Prevention, 2019). It was estimated that CDI caused approximately half a million cases and up to 29,300 deaths annually in the United States (Lessa et al., 2015). Rapid and accurate diagnosis of CDI is essential to ensure timely and effective patient management.

Laboratory testing remains important for the diagnosis of CDI (Gateau et al., 2018). Current confirmatory methods include toxigenic culture (TC), *C. difficile* cytotoxin neutralization assay (CCNA), enzyme immunoassay (EIA) for glutamate dehydrogenase (GDH) or toxins, and nucleic acid amplification tests (NAATs) (O’Horo et al., 2012; Arimoto et al., 2016; Crobach et al., 2016, 2018; Kraft et al., 2019). TC and CCNA are time-consuming and labor-intensive (Crobach et al., 2016). GDH EIAs only serve as screening tools without diagnostic capacity (Arimoto et al., 2016), and toxin EIAs suffer from limited sensitivity in detecting toxins A (TcdA) and B (TcdB) (Crobach et al., 2016). The advent of NAATs has significantly advanced CDI diagnostics, offering shorter turnaround times, simplified procedures, and improved sensitivity and specificity (Barbut et al., 2011; Deshpande et al., 2011; Norén et al., 2011; O’Horo et al., 2012; Surawicz et al., 2013; Crobach et al., 2016). Currently, polymerase chain reaction (PCR) and loop-mediated isothermal amplification (LAMP) are widely adopted (McDonald et al., 2018). Although PCR-based methods, such as the Cepheid Xpert *C. difficile* assay, are highly accurate and routinely employed in clinical laboratories, their dependence on complex instrumentation limits the suitability for point-of-care settings (Mahony et al., 2009). LAMP is an isothermal amplification technique that functions at a constant temperature using basic equipment such as a water or metal bath. However, its specificity is compromised by non-specific amplification (Wang et al., 2015). These limitations highlight the urgent need for novel methods that combine sensitivity, specificity, and operational simplicity for CDI diagnosis.

CRISPR-Cas (clustered regularly interspaced short palindromic repeats-CRISPR-associated proteins) systems have revolutionized modern biology, initially through gene editing due to their efficiency, precision, and simplicity (Cong et al., 2013; Mali et al., 2013). The discovery of *trans*-cleavage activity in Cas12 (Chen et al., 2018), Cas13 (East-Seletsky et al., 2016), and Cas14a (Harrington et al., 2018) has expanded their application to molecular diagnostics. Upon formation of a ternary complex (Cas protein–crRNA/sgRNA–target), Cas proteins mediate both site-specific (*cis*-) cleavage of the target and nonspecific collateral (*trans*-) cleavage of surrounding single-stranded nucleic acids (Li et al., 2018a). In diagnostic assays, cleavage of fluorescent probes releases a fluorophore, generating a detectable signal indicative of target presence (Kellner et al., 2019). Coupling CRISPR-Cas systems with nucleic acid amplification significantly enhances detection sensitivity, as demonstrated by platforms such as SHERLOCK (specific high-sensitivity enzymatic reporter unlocking; RPA-Cas13a) (Gootenberg et al., 2017), HOLMES (one-hour low-cost multipurpose highly efficient system; PCR/RT-PCR-Cas12a) (Li et al., 2018a, b), and DETECTR (DNA endonuclease-targeted CRISPR trans-reporter; RPA-Cas12a) (Chen et al., 2018), each achieving attomolar sensitivity. Moreover, the requirements for protospacer adjacent motif (PAM) recognition and precise base pairing enable CRISPR-based assays to attain single-base resolution (Chen et al., 2025). However, incompatibility between amplification and CRISPR detection remains challenging, primarily due to mismatched reaction temperatures and premature cleavage of amplification products (Lu et al., 2022). Consequently, amplification and detection are typically performed sequentially, complicating the workflow and increasing the risk of aerosol contamination. To address this, platforms such as HOLMESv2 (Li et al., 2019), STOPCovid (SHERLOCK Testing in One Pot) (Joung et al., 2020), CRISPR-SPADE (CRISPR Single Pot Assay for Detecting Emerging Variants of Concern) (Nguyen et al., 2022), and SPLENDID (Single-Pot LAMP-mediated Engineered BrCas12b for Nucleic Acid Detection of Infectious Diseases) (Nguyen et al., 2023) have leveraged thermostable Cas12b variants to enable single-tube detection. Beyond viral targets, LAMP-CRISPR platforms have also been applied to the detection of bacteria such as *Mycobacterium tuberculosis* (Sam et al., 2021), *Klebsiella pneumoniae* (Qiu et al., 2022), *Pseudomonas aeruginosa* (Qiu et al., 2023), and *Salmonella Typhimurium* (Gong et al., 2024). Notably, recent studies have demonstrated that employing non-canonical PAMs can further enhance the performance of one-pot CRISPR assays by reducing *cis*-cleavage activity and minimizing amplification product consumption (Lu et al., 2022). A suboptimal PAM-based Cas12a detection method (sPAMC) achieved 94.2% sensitivity and 100% specificity for SARS-CoV-2 detection within 20 minutes (Lu et al., 2022). Additionally, a one-step RPA-CRISPR detection (ORCD) platform bypassing classical PAM constraints enabled the detection of as few as 0.2 copies/μL of DNA and 0.4 copies/μL of RNA (Lin et al., 2023).

**Figure 1:**
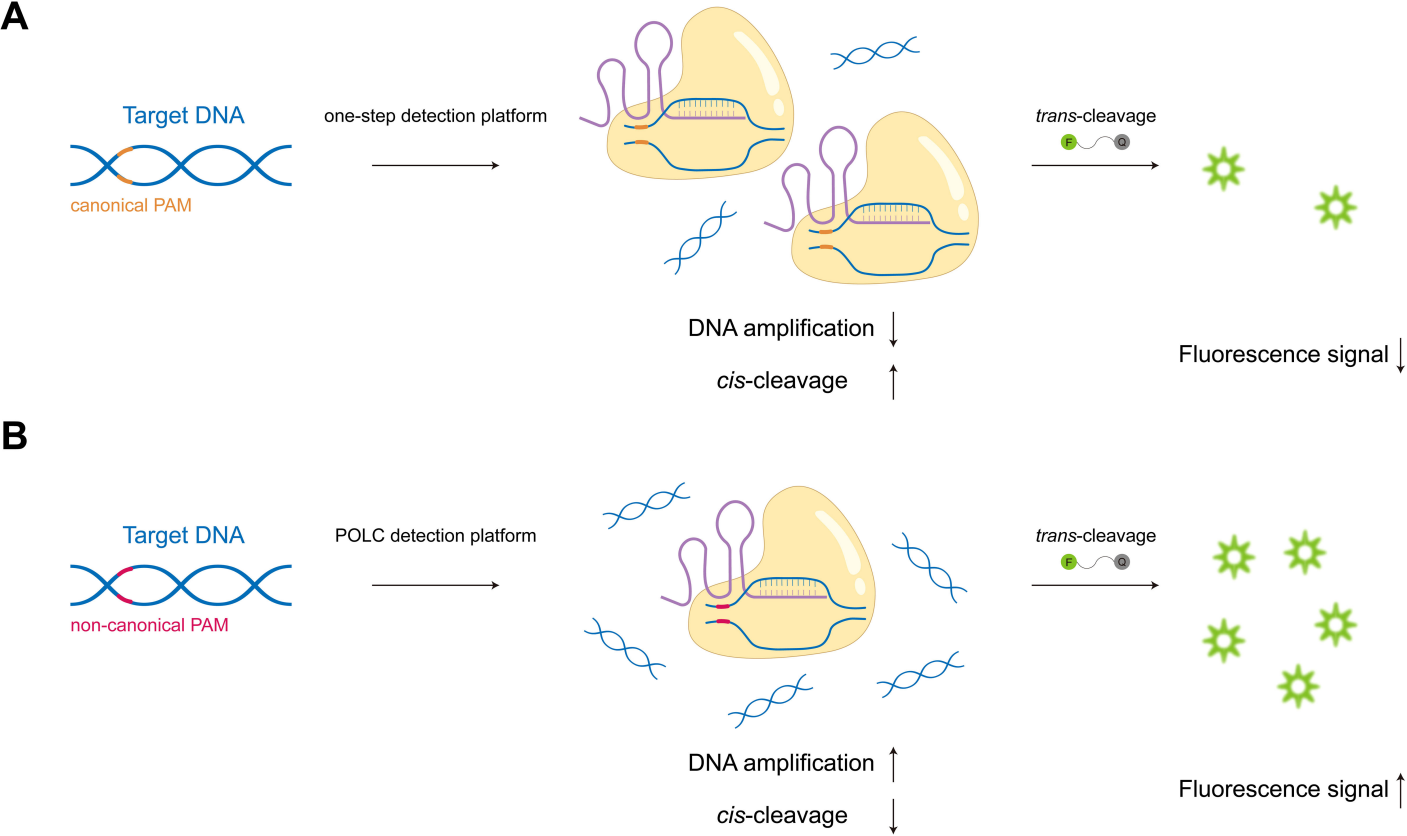
Schematic illustration of one-step CRISPR-based detection platforms under canonical and non-canonical PAM conditions. **(A)** When recognizing canonical PAMs, Cas effectors exhibit high *cis*-cleavage activity towards target nucleic acids, impairing target enrichment and resulting in weak fluorescence signals. **(B)** In the POLC platforms, the use of non-canonical PAMs weakens Cas binding, allowing amplification to dominate the early phase of the reaction. This leads to effective target accumulation and enhances fluorescence signal production.

**Figure 2:**
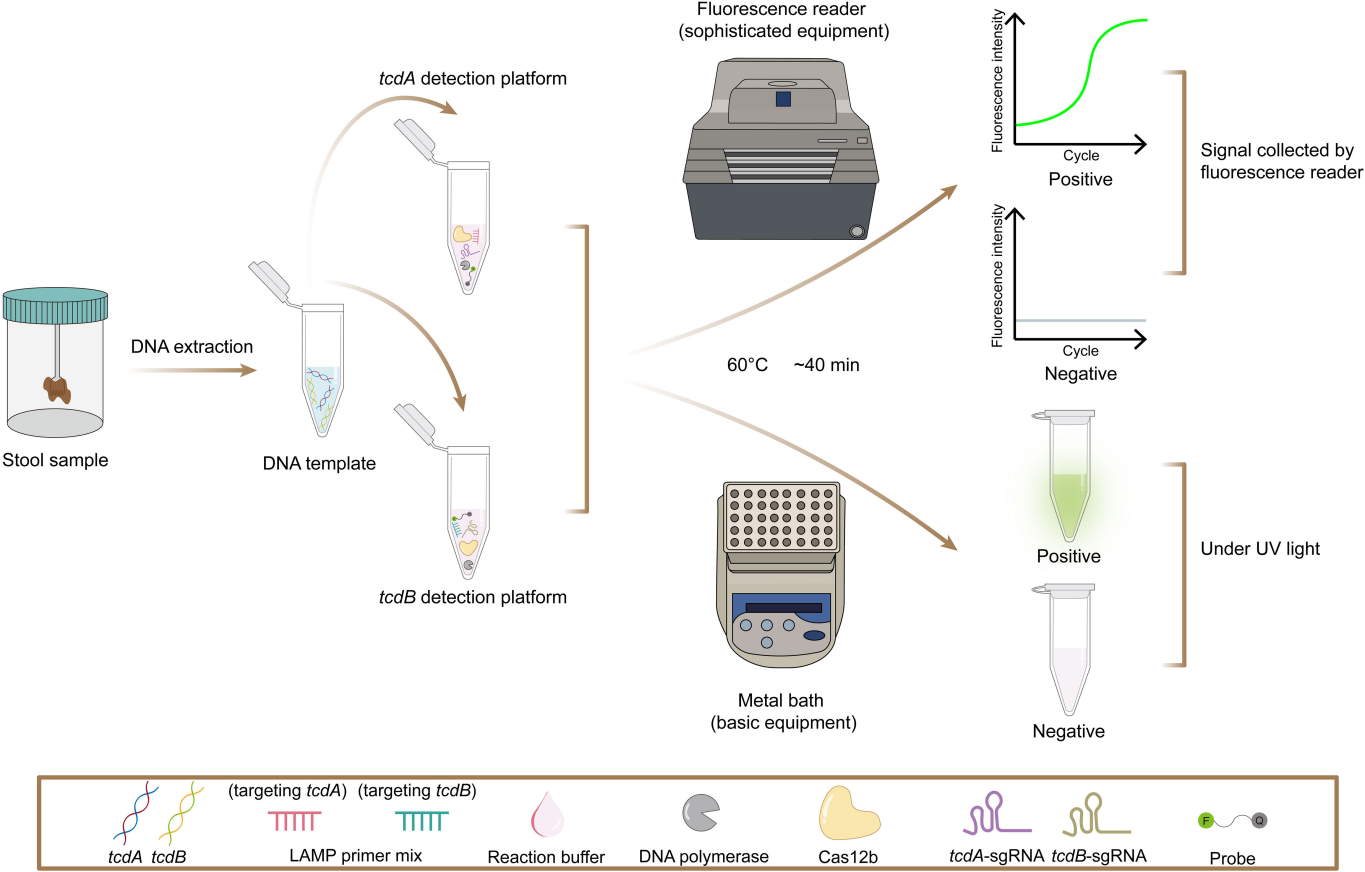
Workflow of the POLC detection platforms targeting *tcdA* and *tcdB*.

In this study, we developed the POLC (PAM-unconventional, One-step LAMP/CRISPR-Cas12b) detection platform, which integrates LAMP with a CRISPR-Cas12b system. Unlike traditional CRISPR-based assays, POLC functions independently of canonical PAM sequences, enhancing target flexibility and simultaneously promoting robust fluorescence signal generation (**Figure 1**). Based on this platform, we established two separate one-step, single-tube assays for the detection of *tcdA* and *tcdB*, respectively. Each assay requires only the addition of sample DNA to a premixed reagent, with results obtainable using a fluorescence plate reader or through direct visualization under UV light, demonstrating applicability in both laboratory and point-of-care settings (**Figure 2**).

## 2 Materials and methods

### 2.1 Reagents and instrumentation

The 10× Isothermal Amplification Buffer, 100 mM MgSO4 Solution, and *Bst* 2.0 Warmstart^®^ DNA Polymerase, were purchased from New England Biolabs Inc. (MA, USA). The dNTP mix (10 mM) was obtained from Hongene Biotech Corp. (Shanghai, China). All nucleotides and additives, including glycine, formamide, and tetramethyl ammonium chloride (TMAC), were sourced from Sangon Biotech Co., Ltd. (Shanghai, China), while betaine and dimethyl sulfoxide (DMSO) were purchased from Aladdin Biochemical Technology Co., Ltd. (Shanghai, China) and Sigma-Aldrich Co. (MO, USA), respectively. The 10× EasyTaq^®^ Buffer was provided by Transgen Biotech. Co., Ltd. (Beijing, China). Salmon sperm DNA solution, SYTO™ 9 dye, and nuclease-free water were purchased from Thermo Fisher Scientific Inc. (MA, USA). *Alicyclobacillus acidiphilus* Cas12b (AapCas12b) and the Cas12b High Yield sgRNA Synthesis and Purification Kit were obtained from TOLO Biotech. Co., Ltd. (Shanghai, China). The 10× SYBR Green DigitalAmp^®^ PCR Kit was purchased from Zhenzhun Bio-Tech. Co., Ltd. (Shanghai, China). The QIAamp^®^ Fast DNA Stool Mini Kit was obtained from Qiagen Co., Ltd. (Shanghai, China), and the *C. difficile toxin A/B* gene detection kit was sourced from Hongweitest Biotechnology Co., Ltd. (Jiangsu, China).

Instruments for *in vitro* transcription included a GE series thermal cycler (Bio-Gener Technology Co., Ltd, Hangzhou, China) for annealing and incubation, and a NanoDrop 2000 spectrophotometer (Thermo Fisher Scientific Inc., MA, USA) for sgRNA quantification. The AccuMini series digital PCR system was purchased from Zhenzhun Bio-Tech. Co., Ltd. (Shanghai, China). LAMP and POLC assays were performed on the LineGene 9600 Plus (Bioer Technology Co., Ltd, Hangzhou, China). The qPCR for clinical validation was conducted on the QuantStudio™ 5 Real-Time PCR Instrument (Applied Biosystems Inc., CA, USA).

### 2.2 LAMP primer design and template preparation

LAMP primers targeting the reference sequences of *tcdA* (Gene ID: 66353160) and *tcdB* (Gene ID: 66353157) were designed using the NEB^®^ LAMP Primer Design Tool (https://lamp.neb.com/) and PrimerExplorer version 5 software (http://primerexplorer.jp/lampv5e/index.html). To check sequence conservation within the primer design regions, ClustalW multiple sequence alignment (Bioedit Sequence Alignment Editor software, v.7.2.5) was performed on *tcdA* and *tcdB* sequences retrieved from the National Center for Biotechnology Information (NCBI) nucleotide database. The analysis revealed that the targeted regions of *tcdA* were highly conserved, whereas those of *tcdB* exhibited relatively lower conservation. BLAST (Basic Local Alignment Search Tool) analysis further confirmed that the amplification regions and adjacent zones of the *tcdA* and *tcdB* reference sequences shared high homology with *C. difficile* 630, designated as *tcdA* and *tcdB1*, respectively. Two additional *tcdB* variants homologous to ribotype 027 (RT027) and 078 (RT078) strains were identified and named *tcdB2* and *tcdB3*, respectively. The sequences of *tcdA*, *tcdB1*, *tcdB2*, and *tcdB3* were synthesized and cloned into pUC57 vectors to generate four types of standard plasmids, which served as template DNA. Detailed information on the template sequences was shown in **Supplementary Figure 1**.

To minimize false negatives, primer positions were slightly adjusted to target conserved regions, and degenerate bases were introduced at mutation sites. Three primer sets were designed for each *tcdB* plasmid (*tcdB1*-P1 to P3, *tcdB2*-P1 to P3, *tcdB3*-P1 to P3). Primer sets *tcdB1*-P3, *tcdB2*-P3, and *tcdB3*-P3 were generated by modifying the F2 region of the forward inner primer (FIP) from *tcdB1*-P2, *tcdB2*-P2, and *tcdB3*-P2, respectively, to further improve sequence conservation. For *tcdA*, three primer sets (*tcdA*-P1 to P3) were designed. Primer specificity was verified by BLAST analysis, and sequences were provided in **Supplementary Tables 1**, **2**.

### 2.3 Accurate quantification of plasmid templates

The copy number of the serially diluted plasmids used in subsequent experiments was quantified by 10× SYBR Green DigitalAmp^®^ PCR Kit according to the operation instructions. Each 20 μL reaction contained 2 μL of 10× SYBR Green PCR Master Mix, 1 μL of PCR primer mix, 2 μL of standard plasmid, and 15 μL of nuclease-free water. The thermal cycling procedure was as follows: 95 °C for 10 minutes, followed by 45 cycles of 95 °C for 30 seconds and 58 °C for 45 seconds. After sample loading, amplification, and chip reading, data were finally analyzed by digital PCR analysis software. The primers used in this reaction were listed in **Supplementary Table 3**.

### 2.4 LAMP primer and additive screening

To screen for the most efficient LAMP primers, *tcdA*-P1 to P3 were evaluated for the amplification of *tcdA*-plasmid. Similarly, *tcdB1*-P1 to P3, *tcdB2*-P1 to P3, and *tcdB3*-P1 to P3 were assessed using their corresponding *tcdB1*-, *tcdB2*-, and *tcdB3*-plasmid. Primer sets demonstrating superior amplification efficiency for each *tcdB* variant were subsequently subjected to cross-validation by amplifying all three *tcdB* plasmids. Sets achieving consistently high and stable amplification across all templates were selected for further development. LAMP reactions were performed following the standard protocol provided by NEB^®^ (https://www.neb.cn/zh-cn/protocols/2014/12/29/typical-lamp-protocol-m0538) in a final volume of 25 μL. Each reaction included 5 μL of plasmid diluted in salmon sperm DNA solution (to reduce loss during gradient dilution) as the positive control, or 5 μL of salmon sperm DNA alone as the negative control. SYTO™ 9 (1 μM) was added as the fluorescent dye, and real-time amplification was monitored on the LineGene 9600 Plus via the FAM channel (470–525 nm).

To improve nucleic acid amplification performance, several additives previously recognized as PCR enhancers were tested at various final concentrations: betaine (0/200/400/600 mM), DMSO (0/2/4/6%), formamide (0/1/2/3%), TMAC (0/10/20/30 mM), and glycine (0/240/360/480 mM). Betaine and DMSO function by reducing secondary structure formation in GC-enriched regions, thereby preventing DNA polymerase dissociation from the template (Henke et al., 1997; Choi et al., 1999; Jensen et al., 2010). Formamide and TMAC help minimize non-specific priming events (Sarkar et al., 1990; Jang and Kim, 2022). Notably, recent studies have shown that glycine can enhance the sensitivity of nucleic acid amplification and promote one-pot CRISPR-based detection (Joung et al., 2020; Ooi et al., 2021). Therefore, it was also included for evaluation and comparison with the other additives. All LAMP reactions were conducted at 65°C, with fluorescence signals collected once per minute over 25 cycles.

### 2.5 POLC detection platforms

After the establishment of LAMP systems, 20 bp conserved regions within the amplification loci of *tcdA* and *tcdB* were selected as target sequences for sgRNA design. To develop the POLC detection platforms, non-canonical protospacer adjacent motifs (PAMs) were employed to attenuate the *cis*-cleavage activity of Cas12b. Transcription templates were prepared in 20 μL reaction mixtures containing 1× EasyTaq^®^ Buffer, 2 μM T7 promoter (5’-TAATACGACTCACTATAGG-3’), 2 μM target antisense polynucleotide, and nuclease-free water. Annealing was performed on a thermal cycler (95°C for 5 minutes, cooling to 25°C at 0.1°C/s, followed by incubation at 25°C). *In vitro* transcription and sgRNA purification were carried out using the Cas12b High Yield sgRNA Synthesis and Purification Kit. The synthesized sgRNAs were quantified by NanoDrop and diluted to the working concentration of 10 μM. All sgRNA sequences used in this study were listed in **Supplementary Table 3**.

The POLC detection platforms integrated LAMP with non-canonical CRISPR-Cas12b systems. On the basis of the LAMP components described above, sgRNAs and AapCas12b were added to the mixture, while SYTO™ 9 was replaced with a fluorescence-quenched single-stranded DNA (ssDNA) probe, designated 8C-FQ (**Supplementary Table 3**). The initial concentrations of sgRNAs, AapCas12b, and 8C-FQ were set at 500 nM. Template volume was adjusted to 1 μL (quantified plasmids for the positive groups, and salmon sperm DNA for the negative control). The preliminary reaction temperature was maintained at 60°C. The screening of sgRNA was conducted under varying Mg^2+^ concentrations (8–12 mM in 1 mM increments) and dNTP concentrations (0.8-1.4 mM in 0.2 mM increments), which were systematically cross-tested.

To enhance detection efficacy, key reaction parameters were optimized, including the concentrations of AapCas12b, sgRNAs, fluorescent probes, and glycine, as well as the reaction temperature. AapCas12b was examined at concentrations of 125, 250, 500, and 750 nM, with sgRNA concentrations adjusted to achieve molar ratios of 1:1, 1:1.5, and 1:2 (AapCas12b:sgRNA). Probe concentrations were evaluated at 250, 500, 750 nM, and 1 μM. Glycine was tested at concentrations of 0, 240, 360, 480, 600, and 720 mM. Although LAMP typically operates at 60-65°C, AapCas12b exhibits reduced enzymatic activity above 60°C (Notomi et al., 2000; Joung et al., 2020). Therefore, temperature optimization was conducted near 60°C. For the *tcdA* platform, reaction temperatures of 57, 58.2, 59, 60, and 61 °C were screened. For the *tcdB* platform, the tested temperatures were 58, 59.2, 60, 61, and 62°C. The LineGene 9600 Plus instrument imposes a fixed temperature gradient, resulting in non-uniform intervals.

### 2.6 Sensitivity and specificity of the POLC detection platforms

Plasmids quantified by digital PCR were serially diluted and used as template DNA to assess the limit of detection (LoD) of the POLC platforms targeting *tcdA* and *tcdB*, respectively. Each dilution level was tested in eight replicates, with salmon sperm DNA serving as the negative control. To evaluate the portability and visual detection performance of the POLC platforms, the prepared reaction mixtures were incubated in a metal bath at 60°C for approximately 40 minutes, followed by exposure to UV light. Results were interpreted based on color changes visible to the naked eye, and the LoD for visual detection was determined.

The specificity of the POLC detection platforms was validated using nucleic acids extracted from several common opportunistic pathogens in the human intestinal tract, including *C. difficile*, *Klebsiella pneumoniae*, *Pseudomonas aeruginosa*, *Escherichia coli*, *Staphylococcus aureus*, *Enterococcus faecalis*, *Proteus mirabilis*, and *Bacteroides fragilis*. Strain details were provided in **Supplementary Table 4**. All nucleic acids were extracted by the conventional boiling method. Each pathogen was tested in triplicate, with nuclease-free water serving as the negative control.

### 2.7 Clinical validation

To verify the practicability of the POLC detection platforms, stool samples were collected from patients with suspected CDI at Huashan Hospital, Fudan University—a teaching hospital with multiple campuses in Shanghai, China. The enrolled patients met all of the following criteria: (1) age ≥18 years; (2) receipt of antibiotic therapy or chemotherapy within the past 60 days; (3) diarrhea occurring three or more times per day; and (4) stool classified as Bristol types 5-7. Exclusion criteria included: (1) chronic diarrhea; (2) use of laxatives within three days prior to symptom onset; and (3) diarrhea with a known cause, such as lactose intolerance. The study was approved by the Institutional Review Board of Huashan Hospital (20230737). All samples were collected after obtaining informed consent and were stored at -80°C.

Fecal DNA was extracted using the QIAamp^®^ Fast DNA Stool Mini Kit according to the manufacturer’s instructions, and was used as the template for both the POLC detection platforms and qPCR. The qPCR was conducted on the QuantStudio™ 5 Real-Time PCR system using a *C. difficile toxin A/B* gene detection kit, which served as the reference method. Each qPCR reaction had a total volume of 32 μL, comprising 25 μL of nucleic acid reaction buffer, 2 μL of internal control, and 5 μL of sample DNA (or Buffer ATE for the negative control). The thermal cycling protocol was as follows: 50°C for 2 minutes, 95°C for 5 minutes, followed by 40 cycles of 95°C for 15 seconds and 55°C for 30 seconds.

### 2.8 Data visualization and statistical analysis

Data visualization and statistical analysis were performed by GraphPad Prism 10 (Graphpad Software Inc., CA, USA), OriginPro 2021 (OriginLab Corp., MA, USA), Adobe Illustrator 2024 (Adobe Systems Inc., CA, USA), and SPSS 29.0 (IBM Corp., NY, USA). McNemar’s two-sided test was used to evaluate statistical differences between the results obtained from the POLC detection platforms and qPCR. A *P* value < 0.05 was considered statistically significant. Consistency between these two methods was assessed by the Kappa statistic, interpreted according to the following scale: 0.00-0.20 (slight), 0.21-0.40 (fair), 0.41-0.60 (moderate), 0.61-0.80 (substantial), and 0.81-1.00 (almost perfect) (Landis and Koch, 1977).

## 3 Results

### 3.1 Design of the LAMP systems

**Figure 3:**
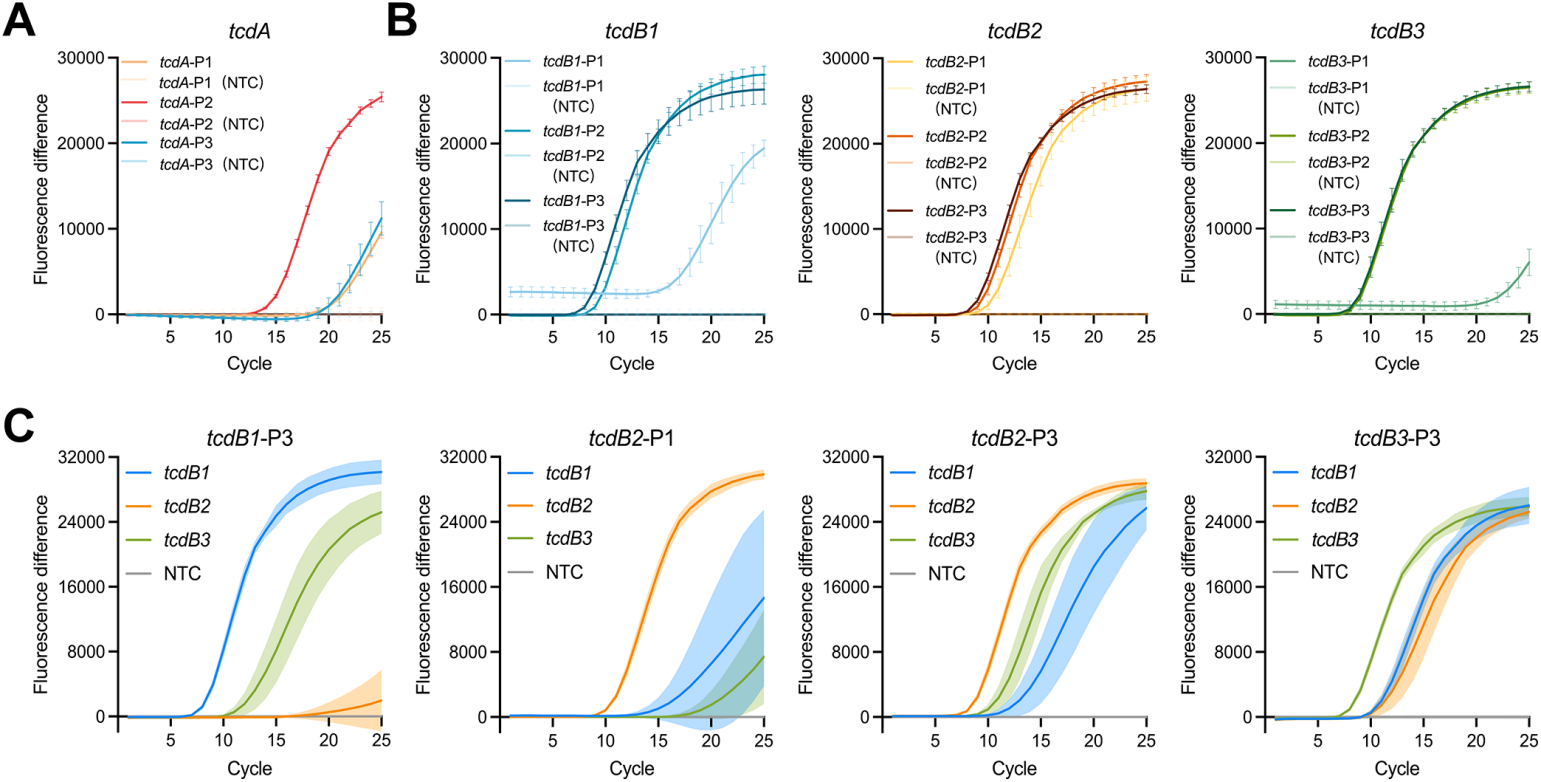
LAMP primer screening. **(A)** Screening of primers targeting *tcdA* (n = 6). Positive control, *tcdA*-plasmid. Negative control, salmon sperm DNA. **(B)** Initial screening of primers targeting *tcdB* (n = 6). Positive control, *tcdB1*-, *tcdB2*-, and *tcdB3*-plasmid. Negative control, salmon sperm DNA. **(C)** Final screening of primers targeting *tcdB* (n = 4). Positive control, *tcdB1*-, *tcdB2*-, and *tcdB3*-plasmid. Negative control, salmon sperm DNA. Fluorescence difference was calculated by subtracting the fluorescence intensity of the negative control from that of the positive control at each cycle. Error bars and shaded areas represent mean ± SD. NTC, no template control.

To develop a highly sensitive detection platform based on CRISPR-Cas systems, it was essential to integrate nucleic acid amplification methods. Among these, LAMP is a well-established technique capable of rapidly generating large amounts of target DNA through self-cycling mechanisms (Notomi et al., 2000). We conducted the reactions using specifically designed primers targeting the *tcdA*-, *tcdB1*-, *tcdB2*-, and *tcdB3*-plasmid. The quantitative results for each plasmid type obtained from digital PCR were shown in **Supplementary Figure 2**. The optimal LAMP primers were selected based on time-to-detection, fluorescence intensity, and signal reproducibility. In this context, time-to-detection refers to the point at which the fluorescence signal exceeds the baseline threshold, marking the initiation of exponential amplification. As shown in **Figure 3A**, the system utilizing the primer *tcdA*-P2 could detect the *tcdA*-plasmid within 15 cycles (18 minutes) and generate the highest fluorescence intensity during the detection process. As shown in **Figure 3B**, the primers *tcdB1*-P3, *tcdB2*-P1, *tcdB2*-P3, and *tcdB3*-P3, which targeted highly conserved regions of the template sequences, exhibited a shorter time-to-detection and higher fluorescence intensity during the amplification of their corresponding plasmids. To minimize false negatives resulting from incomplete primer-template matching, the aforementioned efficient primers (i.e. *tcdB1*-P3, *tcdB2*-P1, *tcdB2*-P3, and *tcdB3*-P3) were used to amplify all three *tcdB* plasmids for versatility testing. As shown in **Figure 3C**, the primer *tcdB1*-P3 exhibited low efficiency in amplifying the *tcdB2*-plasmid, producing only a weak fluorescence signal at 25 cycles. Likewise, the primer *tcdB2*-P1 significantly delayed time-to-detection when used for *tcdB1*- and *tcdB3*-plasmid amplification. Both systems employing the primers *tcdB2*-P3 and *tcdB3*-P3 successfully detected the *tcdB1*-, *tcdB2*-, and *tcdB3*-plasmid within 15 cycles (18 minutes). However, amplification of the *tcdB1*-plasmid with the primer *tcdB2*-P3 showed poor reproducibility, whereas the primer *tcdB3*-P3 exhibited high stability across all three plasmids. Finally, the primers *tcdA*-P2 (**Figure 3A**) and *tcdB3*-P3 (**Figure 3C**) were chosen to develop POLC detection platforms targeting *tcdA* and *tcdB*, respectively. The location of selected primers was displayed in **Supplementary Figure 1**.

To further improve the efficiency of LAMP, several additives were evaluated. As shown in **Supplementary Figure 3**, glycine was more effective in reducing time-to-detection and enhancing fluorescence intensity. Among the tested concentrations, the system supplemented with 480 mM glycine exhibited the highest fluorescence intensity at the end of the reaction, indicating its potential to optimize the amplification yield under this condition. However, most other additives showed no significant improvement in reaction efficiency and, in some cases, exhibited even inhibitory effects. Therefore, 480 mM glycine was chosen for subsequent experiments.

### 3.2 Establishment of the POLC detection platforms

**Figure 4:**
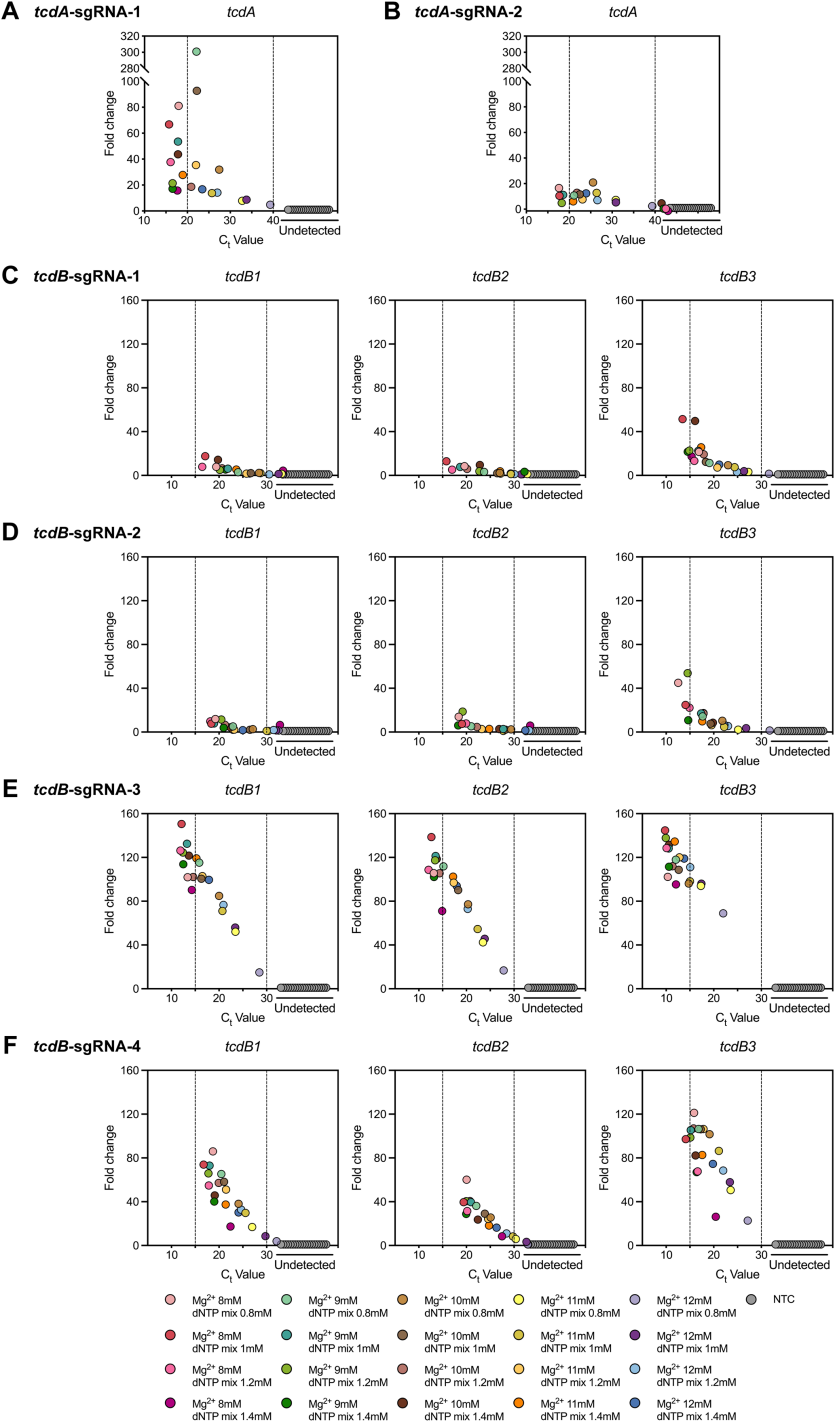
Establishment of the POLC detection platforms using different sgRNAs and various concentrations of Mg^2+^ and dNTP mix. **(A, B)** Responses to the usage of *tcdA*-sgRNA-1 and *tcdA*-sgRNA-2, respectively. Positive control, *tcdA*-plasmid. Negative control, salmon sperm DNA. **(C-F)** Responses to the usage of *tcdB*-sgRNA-1, *tcdB*-sgRNA-2, *tcdB*-sgRNA-3, and *tcdB*-sgRNA-4 in order. Positive control, *tcdB1*-, *tcdB2*-, and *tcdB3*-plasmid. Negative control, salmon sperm DNA. Fold change was defined as the ratio of the fluorescence intensity difference between the last and first cycles in the positive control to that in the negative control. NTC, no template control.

In CRISPR-Cas12b-based nucleic acid testing, the sgRNA not only ensures specificity by strictly base-pairing with target sequences, but also guides Cas12b in locating, recognizing, and binding to the targets (Weng et al., 2023). Cas12b is an endonuclease-capable effector that cleaves both target DNA and non-specific ssDNA via its RuvC domain (Yan et al., 2019). Notably, Mg^2+^ is essential for activating the RuvC domain (Zetsche et al., 2015; Swarts et al., 2017; Dai et al., 2019). Furthermore, it influences DNA polymerase activity during nucleic acid amplification by interacting with dNTPs and nucleic acid backbones (Batra et al., 2006; Xia et al., 2011). Consequently, this study established the POLC detection platforms by screening sgRNAs and the concentrations of Mg^2+^ and dNTP mix. As shown in **Figure 4**, the optimal reaction efficiency was achieved with *tcdA*-sgRNA-1 and *tcdB*-sgRNA-3 in the construction of *tcdA* and *tcdB* POLC detection platforms, respectively. This improvement was evidenced by a lower C_t_ value and a greater fold change in fluorescence intensity. The distribution of the preferential sgRNAs was shown in **Supplementary Figure 1**. Additionally, the *tcdA* POLC detection platform incorporating *tcdA*-sgRNA-1 exhibited superior performance at Mg^2+^ and dNTP mix concentrations of 8 mM/0.8 mM, 8 mM/1 mM, and 9 mM/1 mM. Similarly, the *tcdB* POLC detection platform with *tcdB*-sgRNA-3 exhibited improved efficiency at Mg^2+^ and dNTP mix concentrations of 8 mM/1 mM, 8 mM/1.2 mM, 9 mM/1 mM, 9 mM/1.2 mM, and 10 mM/1.4 mM.

### 3.3 Optimization of the POLC detection platforms

To optimize the POLC detection platforms, firstly, the concentrations of Mg^2+^ and dNTP mix, corresponding to the points near the upper left corner with better performance (**Figures 4A, E**), were selected for repetition. As shown in **Supplementary Figure 4A**, the fluorescence change of the *tcdA* POLC detection platform was relatively low. Considering its potential impact on the platform’s sensitivity, the configuration with a higher ordinate value on the left was preferred, given the similar C_t_ values. As a result, the concentrations of Mg^2+^ and dNTP mix were set to 8 and 0.8 mM, respectively. The *tcdB* POLC detection platform, targeting all three plasmids (i.e. *tcdB1*-, *tcdB2*-, and *tcdB3*-plasmid), performed well under conditions of 8 mM Mg^2+^ and 1 mM dNTP mix, with particularly high efficiency in detecting the most common sequence *tcdB1* (**Supplementary Figure 4A**).

Secondly, the amounts of sgRNAs and AapCas12b were determined. The results showed that the fluorescence curves for AapCas12b at concentrations of 125 and 250 nM closely mirrored those of the negative control, with all curves clustering near the abscissa axis. Considering time-to-detection, fluorescence intensity, and cost, the optimal conditions were achieved with AapCas12b and sgRNA concentrations of 500 nM for the *tcdA* POLC detection platform and 750 nM for the *tcdB* POLC detection platform (**Supplementary Figure 4B**).

Thirdly, the optimal reaction temperature was ascertained. For the *tcdA* POLC detection platform, reactions conducted at 57 and 61°C failed to generate detectable signals across all replicates and were therefore excluded from further analysis. Among the remaining conditions (58.2, 59, and 60°C), statistical analysis indicated that reactions at 60°C yielded the lowest C_t_ values, meaning that the *tcdA* assay produced positive results most rapidly at this temperature (**Supplementary Figure 4C**). For the *tcdB* POLC platform, although the C_t_ values at 60 and 61°C were not significantly different (**Supplementary Figure 4C**), the endpoint fluorescence intensity was higher at 60°C (**Supplementary Figure 4D**), supporting its selection as the preferred temperature.

Next, the ssDNA probe concentration needed to be decided. As shown in **Supplementary Figure 4E**, the efficiency of fluorescence signal generation was improved at higher probe concentrations. However, further increasing the concentration might lead to an elevated fluorescence background in the reaction system lacking target nucleic acids, as well as incur unnecessary cost increases. Thus, the probe of 1 μM was chosen for both *tcdA* and *tcdB* POLC detection platforms.

Finally, glycine concentrations were tested. For the *tcdA* POLC detection platform, the reaction without glycine failed to produce positive results within 40 cycles (**Supplementary Figure 4F**). As the glycine concentration increased, both the time-to-detection was shortened and the fluorescence intensity was enhanced (**Supplementary Figure 4F**). Hence, 720 mM glycine was selected for the *tcdA* POLC detection platform. Regarding the *tcdB* POLC detection platform, only weak signals were generated in the absence of glycine, while the reaction efficiency markedly improved with glycine addition (**Supplementary Figure 4F**). Due to the similar efficiencies observed with 480, 600, and 720 mM glycine, the concentration of 480 mM was preferred to save costs (**Supplementary Figure 4F**).

### 3.4 LoD and specificity of the POLC detection platforms

**Figure 5:**
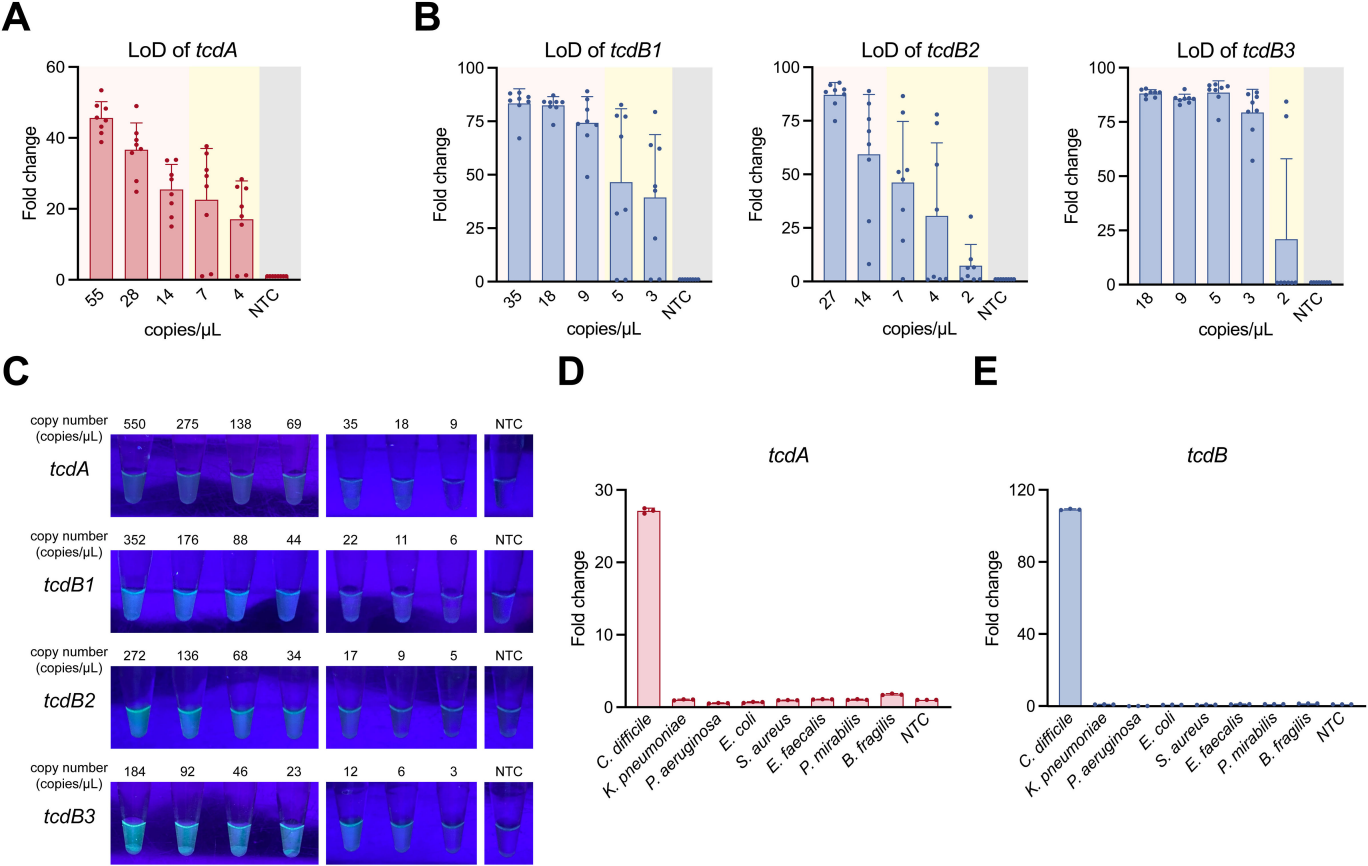
LoD and specificity of the POLC detection platforms. **(A, B)** LoD of the POLC detection platforms targeting the *tcdA*-, *tcdB1*-, *tcdB2*-, and *tcdB3*-plasmid was determined using a qPCR instrument (n = 8). The pink area indicates consistent positive detection results, whereas the yellow area indicates inconsistent results (both positive and negative) at the same plasmid copy number. The gray area represents the negative control (salmon sperm DNA solution). LoD was defined as the lowest template copy number at which the fluorescence intensity of all positive tests reached the detection threshold. Error bars represent mean ± SD. NTC, no template control. **(C)** LoD visualization of the POLC detection platforms targeting the *tcdA*-, *tcdB1*-, *tcdB2*-, and *tcdB3*-plasmid under UV illumination. **(D, E)** The specificity of the POLC detection platforms targeting *tcdA* and *tcdB* (n = 3). Error bars represent mean ± SD. NTC, no template control.

Serial dilutions of *tcdA*-, *tcdB1*-, *tcdB2*-, and *tcdB3*-plasmid were used to evaluate the LoD of the POLC platforms. As shown in **Figures 5A, B**, the lowest template copy numbers at which all eight replicates yielded positive fluorescence signals were approximately 14, 9, 14, and 3 copies/μL for *tcdA*-, *tcdB1*-, *tcdB2*-, and *tcdB3*-plasmid, respectively. In the visual detection assay (**Figure 5C**), color changes were observed when template copy numbers reached or exceeded 18, 11, 17, and 6 copies/μL for *tcdA*-, *tcdB1*-, *tcdB2*-, and *tcdB3*-plasmid, respectively. The LoD values obtained from fluorescence-based and visual readouts were comparable.

Several bacteria were used to examine the specificity of the POLC detection platforms. As shown in **Figures 5D, E**, only the reaction with the nucleic acids extracted from toxigenic *C. difficile* yielded positive results, whereas the reactions involving nucleic acids extracted from the remaining bacteria as well as nuclease-free water corresponded to negative results.

### 3.5 Clinical validation of the POLC detection platforms

**Figure 6:**
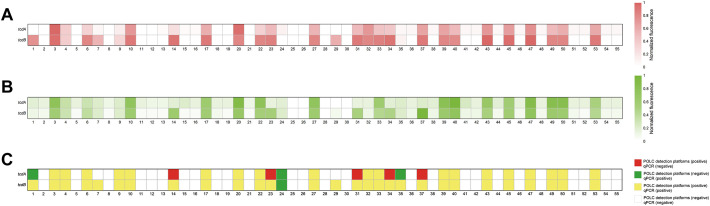
Detection of clinical specimens from patients with suspected CDI by the POLC detection platforms and qPCR. **(A)** Results from clinical samples using the POLC detection platforms. **(B)** Results from clinical samples using qPCR. **(C)** Comparison of qualitative results from clinical samples using the POLC detection platforms and qPCR.

To assess the feasibility of the POLC platforms for detecting *tcdA* and *tcdB* in fecal specimens, 55 clinical stool samples were collected between August 2023 and February 2024. Nucleic acids extracted from these samples served as templates for both the POLC platforms and qPCR. The fluorescence signals generated by the POLC platforms and qPCR were shown in **Figures 6A, B**, respectively. To facilitate direct comparison of *tcdA* and *tcdB* detection by the two methods, the qualitative results were visualized in a heatmap with color-coded blocks: yellow indicates samples positive by both methods; white indicates samples negative by both; red indicates samples positive by the POLC platforms but negative by qPCR; and green indicates samples negative by the POLC platforms but positive by qPCR (**Figure 6C**). In addition, the C_t_ values obtained using the POLC platforms and qPCR for the same samples were presented in **Supplementary Tables 5**, **6**, respectively. Notably, for most positive samples, the C_t_ values derived from the POLC platforms were lower than those from qPCR. Furthermore, by eliminating the need for thermal cycling, the POLC platforms significantly reduced the reaction time to approximately 40 minutes, compared to 73 minutes required for qPCR.

The relationship between the results obtained by the POLC platforms and qPCR was summarized through cross-tabulations (**Tables 1**, **2**). Using qPCR as the reference standard, the sensitivity, specificity, positive predictive value (PPV), and negative predictive value (NPV) of the POLC platform for *tcdA* detection were 86.4% (19/22), 84.8% (28/33), 79.2% (19/24), and 90.3% (28/31), respectively. For *tcdB*, the corresponding values were 96.6% (28/29), 100% (26/26), 100% (28/28), and 96.3% (26/27), respectively (**Table 3**). Additionally, the Kappa statistic was applied to assess the agreement between the two approaches. The Kappa coefficients for *tcdA* and *tcdB* detection were 0.701 (substantial agreement) and 0.964 (almost perfect agreement), respectively (**Table 3**). Although results interpreted by the two methods were not entirely identical in a few cases, McNemar’s test yielded exact *P* values greater than 0.05, indicating no statistically significant difference between the POLC platforms and qPCR (**Table 3**).

**Table 1:** Detection of *tcdA* in clinical samples using POLC and qPCR.

Tests		qPCR		Total
		*tcdA* (+)	*tcdA* (-)	
POLC	*tcdA* (+)	19	5	24
	*tcdA* (-)	3	28	31
Total		22	33	55

**Table 2:** Detection of *tcdB* in clinical samples using POLC and qPCR.

Tests		qPCR		Total
		*tcdB* (+)	*tcdB* (-)	
POLC	*tcdB* (+)	28	0	28
	*tcdB* (-)	1	26	27
Total		29	26	55

**Table 3:** Statistical analysis of clinical sample verification.

Detection platforms	Target	Reference method	Sensitivity	Specificity	PPV	NPV	*P* value	Kappa
POLC	*tcdA*	qPCR	86.4% (19/22)	84.8% (28/33)	79.2% (19/24)	90.3% (28/31)	0.727	0.701
	*tcdB*	qPCR	96.6% (28/29)	100% (26/26)	100% (28/28)	96.3% (26/27)	1.000	0.964

## 4 Discussion

CDI has caught much attention from public health due to its high levels of morbidity and mortality, as well as the large medical expenditure involved (Smits et al., 2016; Zhang et al., 2016; Guh et al., 2020). In clinical settings, the diagnosis of CDI primarily employs PCR-based methods targeting toxin-encoding genes. Despite their excellent sensitivity and specificity, these methods hinge on thermal cycling process, which leads to a strong dependence on sophisticated temperature control equipment and subsequently drives up the detection costs. As a result, the application of such methods in on-site detection is markedly limited. In light of this, there is an urgent need to develop a detection method suitable for POCT or easily implementable in resource-limited areas.

CRISPR-Cas systems have found widespread applications in the detection of nucleic acids for disease diagnosis. When CRISPR-Cas systems are coupled with nucleic acid amplification techniques, a notable enhancement in detection sensitivity can be achieved (Gootenberg et al., 2017; Myhrvold et al., 2018; Teng et al., 2019). However, the limited compatibility between nucleic acid amplification and CRISPR cleavage often necessitates a two-step detection procedure (Lin et al., 2022). In two-pot assays, amplification and CRISPR cleavage are performed in separate tubes, and the transfer of amplification products increases the risk of contamination (Jiang et al., 2023a). In one-pot assays, the two systems are often physically isolated within a single tube and require centrifugation to initiate the CRISPR reaction (Jiang et al., 2023b; Liu et al., 2023). Although this reduces the risk of contamination, it still compromises operational simplicity.

In this study, we developed one-step detection platforms for *tcdA* and *tcdB* in *Clostridioides difficile* by integrating LAMP with CRISPR-Cas12b, which we termed POLC. To address the limited compatibility mentioned above, we optimized the reaction buffer and designed sgRNAs targeting sequences adjacent to non-canonical PAMs. Although isothermal amplification buffers were available for both LAMP and CRISPR cleavage, ion concentrations still needed optimization to meet the requirements of both systems. In one-step CRISPR-based assays, recognition of canonical PAMs by Cas proteins can trigger strong *cis*-cleavage activity, leading to premature degradation of amplification products and delayed fluorescence signal generation. In contrast, non-canonical PAMs reduce such *cis*-cleavage activity, allowing amplification to proceed more efficiently. This facilitates target enrichment and timely activation of Cas-mediated *trans*-cleavage, producing more robust fluorescence signals (Lu et al., 2022). Moreover, the LAMP reaction may generate single-stranded target DNA, enabling PAM-independent Cas activation. As a result, all components could be premixed, supporting the formulation of a POLC reaction buffer suitable for future commercial diagnostic kits.

Through meticulous optimization of components and reaction temperatures, the POLC platforms achieved efficient isothermal operation, with LoDs ranging from 3 to 14 copies/μL when measured using a qPCR instrument, and from 6 to 18 copies/μL when interpreted visually under UV light. Specificity testing revealed no cross-reactivity with other common gut bacteria. In addition to excellent analytical performance, the POLC platforms demonstrated notable cost-effectiveness, with an estimated cost of approximately USD 6.5 per reaction, compared to around USD 10 for commercial qPCR kits and USD 26 for the Cepheid Xpert *C. difficile* assay. Moreover, the isothermal workflow and direct UV-based detection eliminate the need for specialized thermal cyclers and fluorescence monitoring systems, further reducing operational costs. These features make POLC particularly well-suited for POCT.

To assess the clinical applicability of the POLC platforms, 55 stool samples from patients with suspected CDI were analyzed. Including approximately 25 minutes for fecal DNA extraction, the total turnaround time for POLC was about one hour—roughly 30 minutes shorter than that required for qPCR. Notably, positive signals were often detectable much earlier during the reaction process: some appeared as early as 15 minutes, and most were observed within 30 minutes (C_t_ values listed in **Supplementary Table 5**), further demonstrating the platform’s rapidity. In terms of diagnostic performance, the POLC platforms exhibited high sensitivity and specificity, with results comparable to those obtained by qPCR. Although minor discrepancies were observed, statistical analysis revealed no significant differences. These discrepancies may be attributed to sequence variations in the *tcdA* and *tcdB* target regions among different *C. difficile* strains. The diagnostic capability of the *tcdA* POLC assay was slightly lower than that of the *tcdB* assay. This may be due to the initial assumption that the *tcdA* target region was relatively conserved, which led to less rigorous screening of its LAMP primers and sgRNAs. These factors may have adversely affected downstream detection performance.

In addition, several limitations remain to be addressed. First, the *tcdA* and *tcdB* assays were conducted independently, meaning that simultaneous detection of both genes requires two separate reactions, which somewhat compromises workflow simplicity. Second, *C. difficile* also harbors other virulence-associated genes, including *tcdC*, *cdtA*, and *cdtB* (Awad et al., 2014). At present, the detection scope of the POLC platforms remains limited, and future efforts should focus on developing a multiplex assay capable of identifying multiple targets. Finally, incorporating a nucleic acid-free extraction approach may further enhance operational efficiency and reduce overall detection time.

## 5 Conclusion

Our study devised one-step CRISPR-based diagnostic platforms, termed POLC, for the rapid detection of the *tcdA* and *tcdB* genes in *C. difficile*. By integrating LAMP with non-canonical CRISPR-Cas12b systems, the platforms allow direct sample addition to premixed reagents, followed by simple isothermal incubation. Clinical validation demonstrated that the POLC assays achieve high sensitivity and specificity comparable to qPCR. Moreover, by eliminating the need for thermal cycling and real-time fluorescence monitoring systems, the POLC platforms not only shorten detection time but also simplify equipment requirements and reduce costs, highlighting their strong potential for POCT.

## Data Availability

The raw data supporting the conclusions of this article will be made available by the authors, without undue reservation.
