# Supplementary material for: Innovative nucleic acid detection of Clostridioides difficile utilizing the PAM-unconventional, one-step LAMP/CRISPR-Cas12b detection platforms
Source: Front Cell Infect Microbiol. 2025 May 29;15:1594271. doi: 10.3389/fcimb.2025.1594271 (PMC12159041; doi:10.3389/fcimb.2025.1594271)
Supplement: Supplementary file 1 [file DataSheet1.docx]

Supplementary Material

# Supplementary Figures and Tables

## Supplementary Figures

##
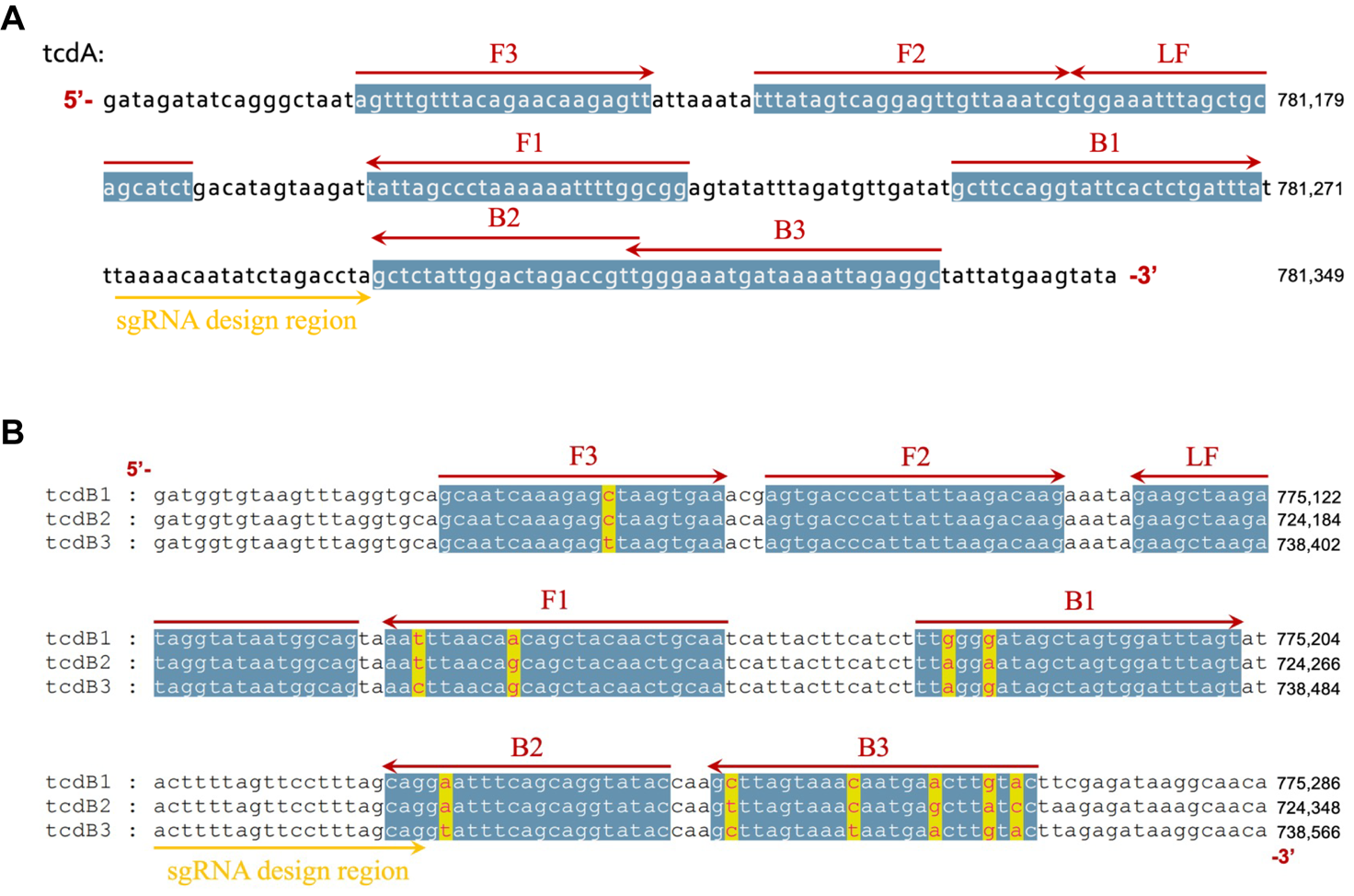
Supplementary Figure 1. Template sequences and the design regions of LAMP primers and sgRNAs. (A) Partial sequence of *tcdA*. (B) Partial sequences of *tcdB1*, *tcdB2*, and *tcdB3*, aligned using MAFFT and visualized with GeneDoc. Blue regions represent the primer design areas used for LAMP amplification. Red labels and arrows indicate the names and orientations of LAMP primers, while yellow arrows denote the locations and directions of the final selected sgRNAs. Sequence variations within the primer design regions are highlighted in yellow.

**
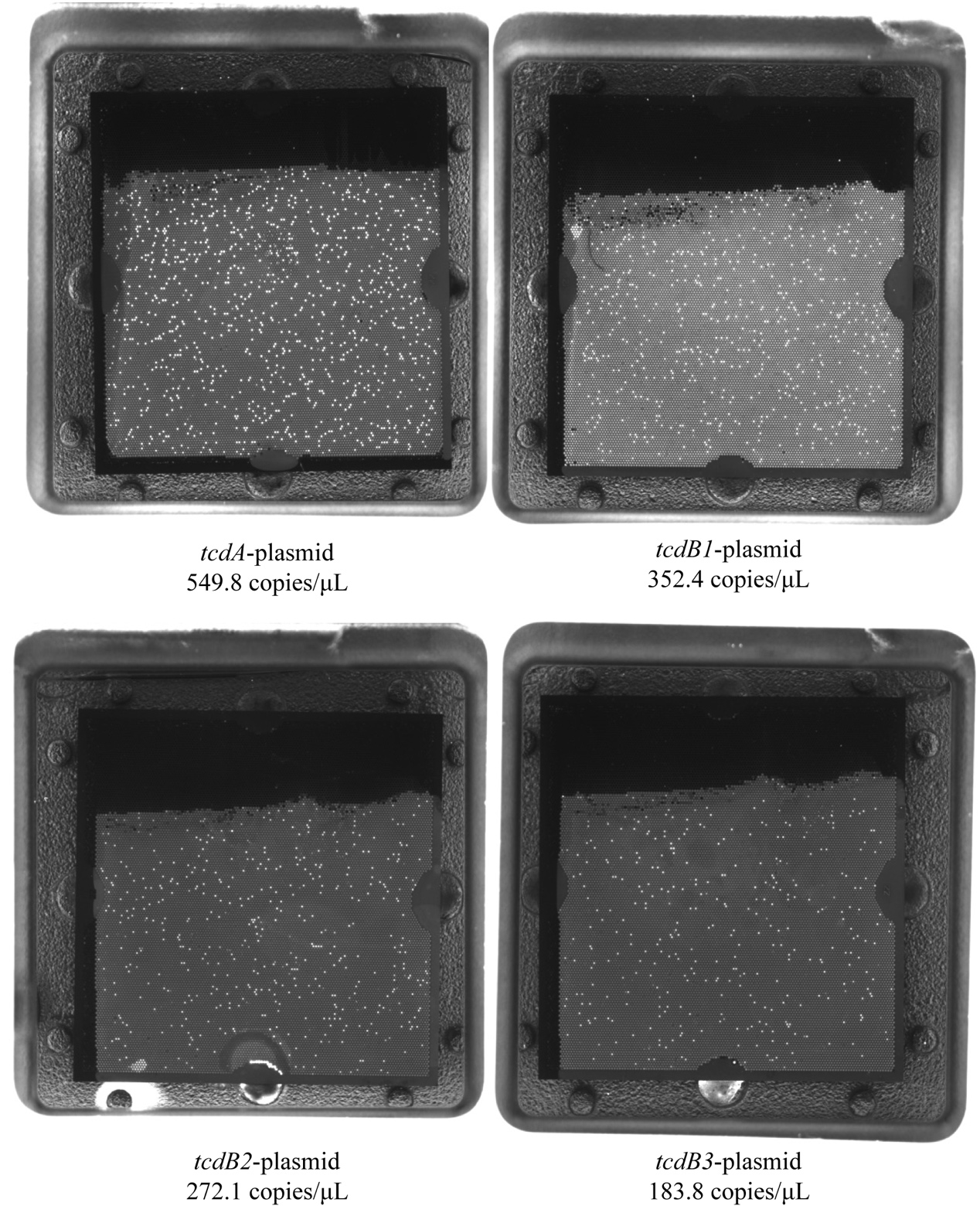
**

**Supplementary Figure 2.** Precise quantification of the plasmids by digital PCR.


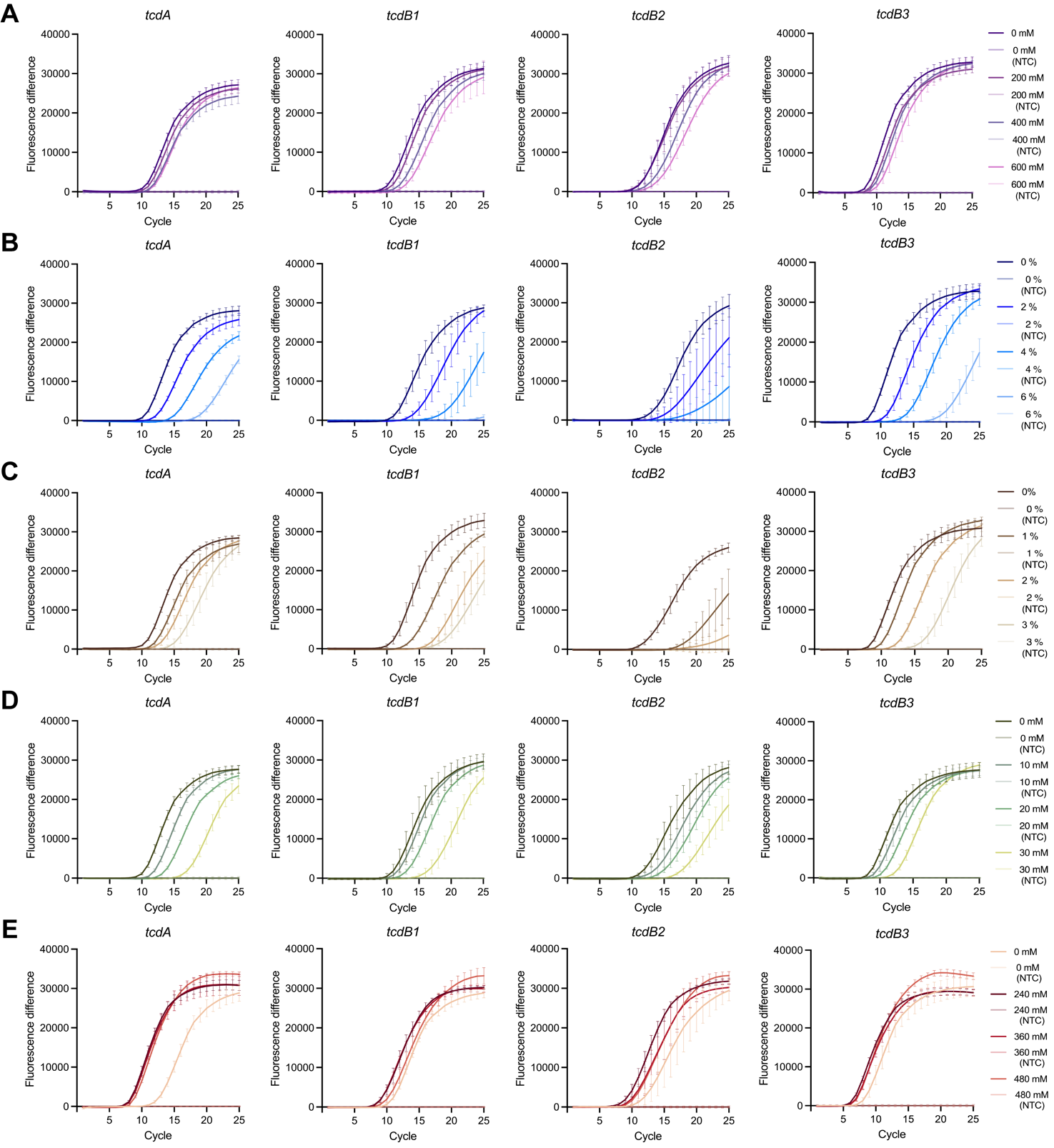


**Supplementary Figure 3.** Optimal additive screening in LAMP. (A-E) The respective use of betaine, DMSO, formamide, TMAC, and glycine as additives for the detection of different templates (n=4). Positive control, *tcdA*-, *tcdB1*-, *tcdB2*-, and *tcdB3*-plasmid. Negative control, salmon sperm DNA solution. Error bars represent mean ± SD. NTC, no template control.


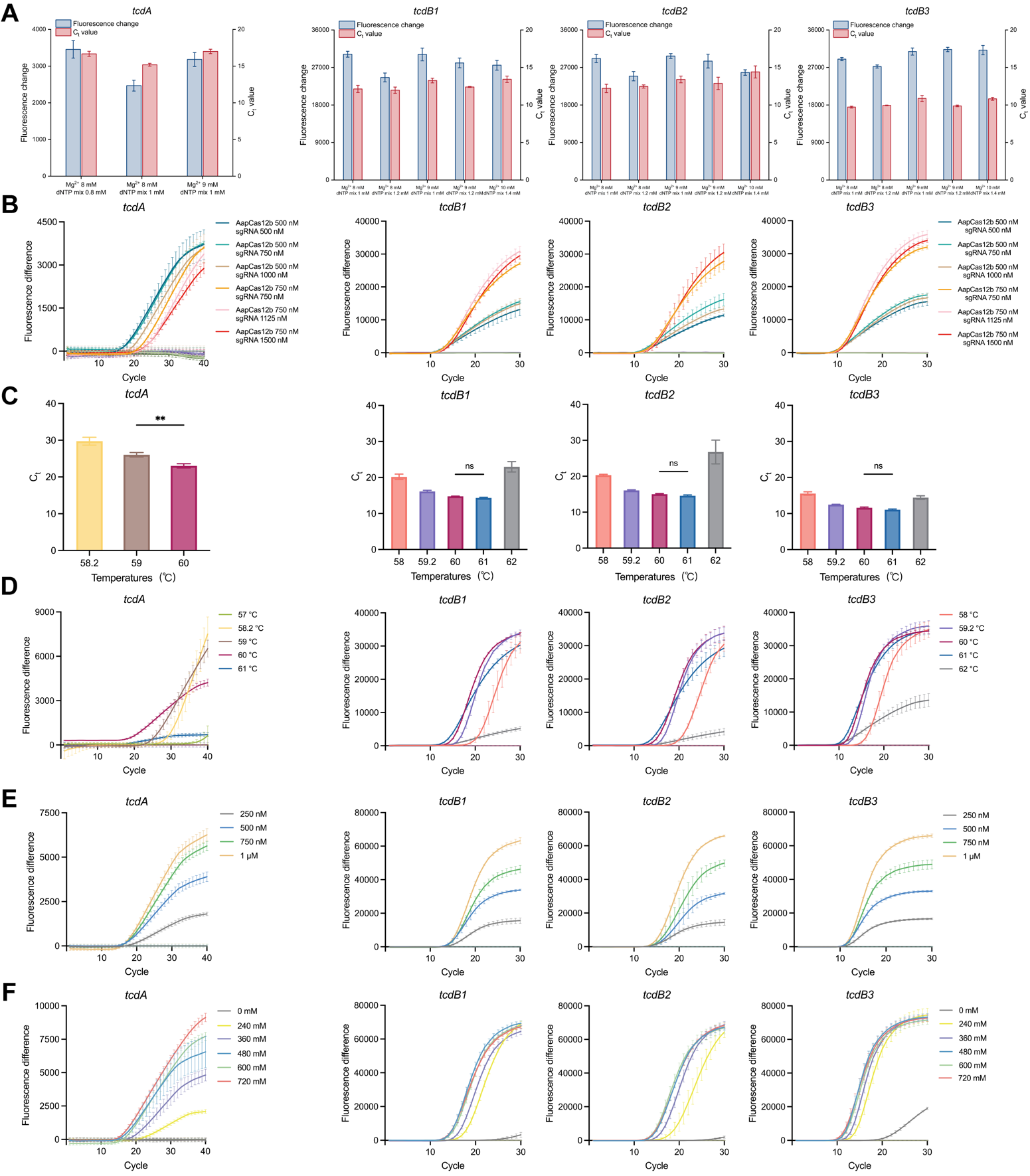


**Supplementary Figure 4.** Optimization of the POLC detection platforms. (A) Determination of the concentrations of Mg^2+^ and dNTP mix preferred in *tcdA* and *tcdB* POLC detection systems (n=4). Fluorescence change represents the difference in fluorescence intensity between the last and first cycles. (B) Screening of the final concentrations of AapCas12b and sgRNAs (n=4 for *tcdA*, n=3 for *tcdB*). (C-D) Confirmation of the optimal reaction temperature (n=4 for *tcdA*, n=3 for *tcdB*). (E) Examination of the concentration of ssDNA reporter (n=4 for *tcdA*, n=3 for *tcdB*). (F) Selection of the glycine concentration in the POLC detection platforms (n=4). Positive control, *tcdA*-, *tcdB1*-, *tcdB2*-, and *tcdB3*-plasmid. Negative control, salmon sperm DNA solution. Error bars represent mean ± SD.

## Supplementary Tables

**Supplementary Table 1.** LAMP primers designed for *tcdA* genes.

| Primer | Type | Sequence (5’-3’) | Length ^a^ |
| --- | --- | --- | --- |
| *tcdA*-P1-F3 | Forward outer primer | GGCTAATAGTTTGTTTACAGAACA | 24 nt |
| *tcdA*-P1-B3 | Backward outer primer | GCCTCTAATTTTATCATTTCCCA | 23 nt |
| *tcdA*-P1-FIP | Forward inner primer | ATACTCCGCCAAAATTTTTTAGGGCGTCAGGAGTTGTTAAATCGTG | 46 nt |
| *tcdA*-P1-BIP | Backward inner primer | GCTTCCAGGTATTCACTCTGATTTAACGGTCTAGTCCAATAGAGC | 45 nt |
| *tcdA*-P1-LF | Loop forward primer | CTTACTATGTCAGATGCTGCAGCTA | 25 nt |
| *tcdA*-P2-F3 ^b^ | Forward outer primer | AGTTTGTTTACAGAACAAGAGTT | 23 nt |
| *tcdA*-P2-FIP | Forward inner primer | CCGCCAAAATTTTTTAGGGCTAATATTTATAGTCAGGAGTTGTTAAATCG | 50 nt |
| *tcdA*-P2-LF | Loop forward primer | AGATGCTGCAGCTAAATTTCCA | 22 nt |
| *tcdA*-P3-F3 | Forward outer primer | ACACCTTAACCCAGCCAT | 18 nt |
| *tcdA*-P3-B3 | Backward outer primer | ATGAAATCATAGTAAGCTGACG | 22 nt |
| *tcdA*-P3-FIP | Forward inner primer | AACATAGAGTTTTCTGCGGTAGCTAGAGTCTGATAATAACTTCACAGA | 48 nt |
| *tcdA*-P3-BIP | Backward inner primer | AGCACCATACTTACAAGTAGGTTTTCTGGACCACTTAAACTTATTGTG | 48 nt |
| *tcdA*-P3-LB | Loop backward primer | ATGCCAGAAGCTCGCTC | 17 nt |

^a^ nt, nucleotide.

^b^ The B3 and BIP of *tcdA*-P2 were the same as those of *tcdA*-P1.

**Supplementary Table 2.** LAMP primers designed for *tcdB* genes.

| Primer | Type | Sequence (5’-3’) | Length |
| --- | --- | --- | --- |
| *tcdB1*-P1-F3 | Forward outer primer | GCTAAGTGAAACRAGTGACC | 20 nt |
| *tcdB1*-P1-B3 | Backward outer primer | TTGCCTTATCTCKAAGTACA | 20 nt |
| *tcdB1*-P1-FIP | Forward inner primer | TGAAGTAATGATTGCAGTTGTAGCTTAGAAGCTAAGATAGGTATAATGGC | 50 nt |
| *tcdB1*-P1-BIP | Backward inner primer | TTGGGGRTAGCTAGTGGATTTAGTTTCATTGTTTACTAAGCTTGGTA | 47 nt |
| *tcdB1*-P1-LB | Loop backward primer | AGCAGGAATTTCAGCAGGTA | 20 nt |
| *tcdB1*-P2-F3 | Forward outer primer | GCAATCAAAGAGYTAAGTGAA | 21 nt |
| *tcdB1*-P2-B3 | Backward outer primer | GTACAAGTTCATTRTTTACTAAGC | 24 nt |
| *tcdB1*-P2-FIP | Forward inner primer | TTGCAGTTGTAGCTGTTGTTAAATTCGAGTGACCCATTATTAAGACA | 47 nt |
| *tcdB1*-P2-BIP | Backward inner primer | TTGGGGRTAGCTAGTGGATTTAGTGTATACCTGCTGAAATTCCTG | 45 nt |
| *tcdB1*-P2-LF | Loop forward primer | CTGCCATTATACCTATCTTAGCTTC | 25 nt |
| *tcdB1*-P3-FIP ^a^ | Forward inner primer | TTGCAGTTGTAGCTGTTGTTAAATTAGTGACCCATTATTAAGACAAG | 47 nt |
| *tcdB2*-P1-B3 ^b^ | Backward outer primer | TTGCTTTATCTCTTAGGATA | 20 nt |
| *tcdB2*-P1-BIP | Backward inner primer | TTAGGAATAGCTAGTGGATTTAGTCTCATTGTTTACTAAACTTGGTA | 47 nt |
| *tcdB2*-P2-B3 ^c^ | Backward outer primer | GGATAAGCTCATTGTTTACTAAAC | 24 nt |
| *tcdB2*-P2-FIP | Forward inner primer | TTGCAGTTGTAGCTGCTGTTAAATTCAAGTGACCCATTATTAAGACA | 47 nt |
| *tcdB2*-P2-BIP | Backward inner primer | TTAGGAATAGCTAGTGGATTTAGTGTATACCTGCTGAAATTCCTG | 45 nt |
| *tcdB2*-P3-FIP ^a^ | Forward inner primer | TTGCAGTTGTAGCTGCTGTTAAATTAGTGACCCATTATTAAGACAAG | 47 nt |
| *tcdB3*-P1-F3 ^b^ | Forward outer primer | GTTAAGTGAAACTAGTGACC | 20 nt |
| *tcdB3*-P1-BIP | Backward inner primer | TTAGGGATAGCTAGTGGATTTAGTTTCATTATTTACTAAGCTTGGTA | 47 nt |
| *tcdB3*-P2-FIP ^d^ | Forward inner primer | TTGCAGTTGTAGCTGCTGTTAAGTTCTAGTGACCCATTATTAAGACA | 47 nt |
| *tcdB3*-P2-BIP | Backward inner primer | TTAGGGATAGCTAGTGGATTTAGTGTATACCTGCTGAAATACCTG | 45 nt |
| *tcdB3*-P3-FIP ^a^ | Forward inner primer | TTGCAGTTGTAGCTGCTGTTAAGTTAGTGACCCATTATTAAGACAAG | 47 nt |

^a^ Only the FIP differed between the second and third sets of primers corresponding to each target. (*tcdB1*-P2/P3, *tcdB2*-P2/P3, *tcdB3*-P2/P3)

^b^ Compared with *tcdB1*-P1, *tcdB2*-P1 shared identical primers except for the B3 and BIP, as did *tcdB3*-P1, but except for the F3 and BIP.

^c^ The F3 and LF of *tcdB2*-P2 were the same as those of *tcdB1*-P2.

^d^ Compared with *tcdB1*-P2, *tcdB3*-P2 shared identical primers except for the FIP and BIP.

**Supplementary Table 3.** Other nucleotides used in this study.

| Type | Sequence (5’-3’) | Length |
| --- | --- | --- |
| *tcdA*-F ^a^ | CAGGGCTAATAGTTTGTTTACAGAACA | 27 nt |
| *tcdA*-R | CAACATCTAAATATACTCCGCCAAAA | 26 nt |
| *tcdB*-F | tacagatgcagccaaagttgt | 21 nt |
| *tcdB*-R1 | gctctttgattgctgcacctaa | 22 nt |
| *tcdB*-R2 | ACTCTTTGATTGCTGCACCTAA | 22 nt |
| 8C-FQ | FAM-CCCCCCCC-BHQ1 | 8 nt |
| T7 promoter | TAATACGACTCACTATAGG | 19 nt |
| *tcdA*-sgRNA-1 | GUCUAGAGGACAGAAUUUUUCAACGGGUGUGCCAAUGGCCACUUUCCAGGUGGCAAAGCCCGUUGAGCUUCUCAAAUCUGAGAAGUGGCACUAAAACAAUAUCUAGACCUA | 111 nt |
| *tcdA*-sgRNA-2 | GUCUAGAGGACAGAAUUUUUCAACGGGUGUGCCAAUGGCCACUUUCCAGGUGGCAAAGCCCGUUGAGCUUCUCAAAUCUGAGAAGUGGCACAGGUCUAGAUAUUGUUUUAA | 111 nt |
| *tcdB*-sgRNA-1 | GUCUAGAGGACAGAAUUUUUCAACGGGUGUGCCAAUGGCCACUUUCCAGGUGGCAAAGCCCGUUGAGCUUCUCAAAUCUGAGAAGUGGCACAUACUUUUAGUUCCUUUAGC | 111 nt |
| *tcdB*-sgRNA-2 | GUCUAGAGGACAGAAUUUUUCAACGGGUGUGCCAAUGGCCACUUUCCAGGUGGCAAAGCCCGUUGAGCUUCUCAAAUCUGAGAAGUGGCACUACUUUUAGUUCCUUUAGCA | 111 nt |
| *tcdB*-sgRNA-3 | GUCUAGAGGACAGAAUUUUUCAACGGGUGUGCCAAUGGCCACUUUCCAGGUGGCAAAGCCCGUUGAGCUUCUCAAAUCUGAGAAGUGGCACACUUUUAGUUCCUUUAGCAG | 111 nt |
| *tcdB*-sgRNA-4 | GUCUAGAGGACAGAAUUUUUCAACGGGUGUGCCAAUGGCCACUUUCCAGGUGGCAAAGCCCGUUGAGCUUCUCAAAUCUGAGAAGUGGCACAGAUGAAGUAAUGAUUGCAG | 111 nt |

^a^ The primers displayed in this table were used for digital PCR, in which *tcdA*-F/R targeted *tcdA-*plasmid, *tcdB*-F/R1 for *tcdB1-* and *tcdB2-*plasmid, *tcdB*-F/R2 for *tcdB3-*plasmid.

**Supplementary Table 4.** Strains used for specificity assessment.

| Bacteria | Type |
| --- | --- |
| *Clostridioides difficile* | Clinical isolates |
| *Pseudomonas aeruginosa* | ATCC 27853 |
| *Klebsiella pneumoniae* | ATCC 700603 |
| *Escherichia coli* | ATCC 25922 |
| *Staphylococcus aureus* | ATCC 29213 |
| *Enterococcus faecalis* | ATCC 29212 |
| *Proteus mirabilis* | Clinical isolates |
| Bacteroides fragilis | ATCC 25285 |

**Supplementary Table 5.** C_t_ values from the POLC platforms in clinical sample detection.

| Sample number | C_t_ value | | Interpretation of results |
| --- | --- | --- | --- |
|  | *tcdA* | *tcdB* |  |
| 1 | / | 22.36 | A^－^B^＋^ |
| 2 | / | / | A^－^B^－^ |
| 3 | 12.87 | 14.40 | A^＋^B^＋^ |
| 4 | 19.03 | 30.91 | A^＋^B^＋^ |
| 5 | / | / | A^－^B^－^ |
| 6 | 19.56 | 21.76 | A^＋^B^＋^ |
| 7 | / | 22.41 | A^－^B^＋^ |
| 8 | / | / | A^－^B^－^ |
| 9 | 29.09 | 28.89 | A^＋^B^＋^ |
| 10 | 12.99 | 14.71 | A^＋^B^＋^ |
| 11 | / | / | A^－^B^－^ |
| 12 | / | / | A^－^B^－^ |
| 13 | / | / | A^－^B^－^ |
| 14 | 18.97 | 17.42 | A^＋^B^＋^ |
| 15 | / | / | A^－^B^－^ |
| 16 | / | / | A^－^B^－^ |
| 17 | 17.4 | 19.97 | A^＋^B^＋^ |
| 18 | / | / | A^－^B^－^ |
| 19 | / | / | A^－^B^－^ |
| 20 | 13.97 | 15.36 | A^＋^B^＋^ |
| 21 | / | / | A^－^B^－^ |
| 22 | 19.9 | 16.51 | A^＋^B^＋^ |
| 23 | 17.03 | 15.64 | A^＋^B^＋^ |
| 24 | / | / | A^－^B^－^ |
| 25 | / | / | A^－^B^－^ |
| 26 | / | / | A^－^B^－^ |
| 27 | 21.13 | 16.04 | A^＋^B^＋^ |
| 28 | / | / | A^－^B^－^ |
| 29 | / | 24.73 | A^－^B^＋^ |
| 30 | / | / | A^－^B^－^ |
| 31 | 16.87 | 15.01 | A^＋^B^＋^ |
| 32 | 15.74 | 17.08 | A^＋^B^＋^ |
| 33 | 16.28 | 17.25 | A^＋^B^＋^ |
| 34 | 16.85 | 15.05 | A^＋^B^＋^ |
| 35 | / | 39.74 | A^－^B^＋^ |
| 36 | / | / | A^－^B^－^ |
| 37 | 16.49 | 13.17 | A^＋^B^＋^ |
| 38 | / | / | A^－^B^－^ |
| 39 | 15.6 | 16.48 | A^＋^B^＋^ |
| 40 | 13.76 | 14.28 | A^＋^B^＋^ |
| 41 | / | / | A^－^B^－^ |
| 42 | / | / | A^－^B^－^ |
| 43 | 15.82 | 16.53 | A^＋^B^＋^ |
| 44 | / | / | A^－^B^－^ |
| 45 | 16.89 | 14.99 | A^＋^B^＋^ |
| 46 | / | / | A^－^B^－^ |
| 47 | 13.46 | 13.30 | A^＋^B^＋^ |
| 48 | / | / | A^－^B^－^ |
| 49 | 13.01 | 13.91 | A^＋^B^＋^ |
| 50 | 15.29 | 16.23 | A^＋^B^＋^ |
| 51 | / | / | A^－^B^－^ |
| 52 | / | / | A^－^B^－^ |
| 53 | 17.60 | 17.30 | A^＋^B^＋^ |
| 54 | / | / | A^－^B^－^ |
| 55 | / | / | A^－^B^－^ |

**Supplementary Table 6.** C_t_ values from qPCR in clinical sample detection.

| Sample number | C_t_ value | | Interpretation of results |
| --- | --- | --- | --- |
|  | *tcdA* | *tcdB* |  |
| 1 | 36.76 | 36.15 | A^＋^B^＋^ |
| 2 | / | / | A^－^B^－^ |
| 3 | 21.65 | 22.01 | A^＋^B^＋^ |
| 4 | 29.83 | 29.35 | A^＋^B^＋^ |
| 5 | / | / | A^－^B^－^ |
| 6 | 31.59 | 29.81 | A^＋^B^＋^ |
| 7 | / | 35.62 | A^－^B^＋^ |
| 8 | / | / | A^－^B^－^ |
| 9 | 37.53 | 34.72 | A^＋^B^＋^ |
| 10 | 26.53 | 26.19 | A^＋^B^＋^ |
| 11 | / | / | A^－^B^－^ |
| 12 | / | / | A^－^B^－^ |
| 13 | / | / | A^－^B^－^ |
| 14 | / | 25.32 | A^－^B^＋^ |
| 15 | / | / | A^－^B^－^ |
| 16 | / | / | A^－^B^－^ |
| 17 | 29.41 | 28.57 | A^＋^B^＋^ |
| 18 | / | / | A^－^B^－^ |
| 19 | / | / | A^－^B^－^ |
| 20 | 23.64 | 23.53 | A^＋^B^＋^ |
| 21 | / | / | A^－^B^－^ |
| 22 | 25.64 | 25.31 | A^＋^B^＋^ |
| 23 | / | 25.32 | A^－^B^＋^ |
| 24 | 36.49 | 32.43 | A^＋^B^＋^ |
| 25 | / | / | A^－^B^－^ |
| 26 | / | / | A^－^B^－^ |
| 27 | 28.05 | 27.31 | A^＋^B^＋^ |
| 28 | / | / | A^－^B^－^ |
| 29 | / | 33.39 | A^－^B^＋^ |
| 30 | / | / | A^－^B^－^ |
| 31 | / | 25.25 | A^－^B^＋^ |
| 32 | 25.47 | 27.05 | A^＋^B^＋^ |
| 33 | 26.74 | 26.04 | A^＋^B^＋^ |
| 34 | / | 24.95 | A^－^B^＋^ |
| 35 | 37.44 | 33.16 | A^＋^B^＋^ |
| 36 | / | / | A^－^B^－^ |
| 37 | / | 23.25 | A^－^B^＋^ |
| 38 | / | / | A^－^B^－^ |
| 39 | 26.71 | 25.96 | A^＋^B^＋^ |
| 40 | 23.68 | 23.13 | A^＋^B^＋^ |
| 41 | / | / | A^－^B^－^ |
| 42 | / | / | A^－^B^－^ |
| 43 | 27.40 | 26.78 | A^＋^B^＋^ |
| 44 | / | / | A^－^B^－^ |
| 45 | 27.98 | 25.83 | A^＋^B^＋^ |
| 46 | / | / | A^－^B^－^ |
| 47 | 21.53 | 21.17 | A^＋^B^＋^ |
| 48 | / | / | A^－^B^－^ |
| 49 | 22.16 | 21.98 | A^＋^B^＋^ |
| 50 | 23.37 | 23.09 | A^＋^B^＋^ |
| 51 | / | / | A^－^B^－^ |
| 52 | / | / | A^－^B^－^ |
| 53 | 28.45 | 27.53 | A^＋^B^＋^ |
| 54 | / | / | A^－^B^－^ |
| 55 | / | / | A^－^B^－^ |
